# 2098: Endogenous reverse transcriptase (LINE-1) in human platelets regulates cell morphology and protein synthetic events

**DOI:** 10.1017/cts.2017.202

**Published:** 2018-05-10

**Authors:** Hansjorg Schwertz, Jesse W. Rowley, Larry W. Kraiss, John V. Moran, Robert A. Campbell, Guy A. Zimmerman, Andrew S. Weyrich, Matthew Thomas Rondina, Gerald G. Schumann, Ulrike Thorack

**Affiliations:** The University of Utah School of Medicine, Salt Lake City, UT, USA

## Abstract

OBJECTIVES/SPECIFIC AIMS: Endogenous RT (eRT) is necessary for the function of retrotransposons, elements that replicate via an RNA intermediate. One source of eRT activity is long interspersed elements (LINE). LINEs, of which there are several subgroups (L1, L2, L3), are retrotransposons that regulate cellular growth and gene expression. Given their diverse and important roles, we hypothesized that L1 elements regulate functional responses in megakaryocytes and platelets; a concept not yet examined in the field. METHODS/STUDY POPULATION: To study eRT in human platelets we used RT activity assays, PCR, and Western blot approaches. Furthermore, we used an RT-inhibitor to dissect the function of eRT, analyzed RT-dependent protein synthetic capacity, and immunoprecipitated RNA-DNA hybrids. RNA-DNA hybrids were also detected by means of ICC and automated analysis using CellProfiler software. RNA-DNA hybrids were validated by PCR and eRT regulated synthesis of target proteins was analyzed using autoradiography and Western blot techniques. Platelets from patients with HIV+ were examined in parallel. RESULTS/ANTICIPATED RESULTS: We identified that highly purified, isolated platelets from healthy subjects possess eRT activity. eRT activity was blocked with the non-nucleoside RT inhibitor nevirapine at concentrations within the therapeutic drug range. L1 elements are bicistronic, containing 2 open reading frames (ORFs), ORF1 and ORF2. Thus, we next identified that human platelets express full-length L1 mRNA containing ORF1 and ORF2. In human platelets, eRT activity was localized to L1 protein containing ribonucleo particles. Platelet eRT reverse transcribed exogenous RNAs, a process inhibited by nevirapine, acting in trans using the 3′-UTR of exogenous mRNAs as a template. To dissect the function of eRT in platelets, we next examined cytoskeletal and protein synthetic events in the presence or absence of nevirapine. Inhibition of eRT in isolated platelets led to characteristically beaded platelets in appearance, strongly resembling bone marrow proplatelets. Parallel increases in platelet reactivity were also observed. As these changes occurred over hours, not minutes, we hypothesized that inhibition of eRT would affect platelet protein synthetic events. Consistent with this, RT inhibition resulted in upregulation of global platelet protein synthesis. We validated upregulation of the synthesis of specific proteins (mitofilin, p-selectin, and L26—a component of the 60S ribosomal subunit essential for mRNA translation). RNA-DNA hybrids, noncanonical nucleic acid structures that regulate gene expression, are enriched in regions where L1 is abundant. RNA-DNA hybrids were present in platelets and expression confirmed via differential digestion of RNAs (eg, with RNase A and RNAse I). Next-generation sequencing of pulled down (eg, immunoprecipitated) platelet RNA-DNA hybrids identified numerous differentially expressed transcripts and we focused on MAP1LC3B (LC3B), a primary regulator of autophagy. Hybrid sequencing results for LC3B were validated using qPCR and we confirmed that LC3B RNA binds to L1-encoded RNA binding protein. Platelets treated with nevirapine had increased total LC3B protein expression. As RT inhibition is an important mechanism to control HIV infection, we examined platelet morphology, activation, and LC3B expression in platelets from HIV+ subjects treated with nevirapine. HIV+ patients treated with RT inhibitors had higher numbers of platelets that were beaded in appearance at baseline, increased platelet reactivity, and differential LC3B expression compared with healthy controls. DISCUSSION/SIGNIFICANCE OF IMPACT: Taken together, these results demonstrate that platelets possess eRT activity that regulates platelet morphology, platelet hyperreactivity, and protein synthetic events. We postulate that eRT activity in platelets may be a new post-transcriptional regulatory checkpoint. Moreover, our findings have implications in HIV+ patients treated with RT inhibitors, where off-target effects may contribute to platelet activation and an increased risk of thrombosis.

